# A Review of Candidate Genes and Pathways in Preeclampsia–An Integrated Bioinformatical Analysis

**DOI:** 10.3390/biology9040062

**Published:** 2020-03-27

**Authors:** Muhammad Aliff Mohamad, Nur Fariha Mohd Manzor, Noor Fadzilah Zulkifli, Nurzaireena Zainal, Abd Rahman Hayati, Asral Wirda Ahmad Asnawi

**Affiliations:** 1 Faculty of Medicine and Health Sciences, Universiti Sains Islam Malaysia, 56100 Kuala Lumpur, Selangor, Malaysia; maliff0108@yahoo.com (M.A.M.); nurfariha@usim.edu.my (N.F.M.M.); nfadzilahz@usim.edu.my (N.F.Z.); drzaireen@usim.edu.my (N.Z.); drhayati@usim.edu.my (A.R.H.); 2 Department of Obstetrics and Gynaecology, Hospital Ampang, 68000 Ampang Jaya, Selangor, Malaysia; 3 Department of Haematology, Hospital Ampang, 68000 Ampang Jaya, Selangor, Malaysia

**Keywords:** placenta, preeclampsia, pregnancy, transcript, sequencing and differentially expressed genes

## Abstract

Preeclampsia is a pregnancy-specific disorder characterized by the presence of hypertension with the onset of either proteinuria, maternal organ or uteroplacental dysfunction. Preeclampsia is one of the leading causes of maternal and fetal mortality and morbidity worldwide. However, the etiopathologies of preeclampsia are not fully understood. Many studies have indicated that genes are differentially expressed between normal and in the disease state. Hence, this study systematically searched the literature on human gene expression that was differentially expressed in preeclampsia. An electronic search was performed through 2019 through PubMed, Scopus, Ovid-Medline, and Gene Expression Omnibus where the following MeSH (Medical Subject Heading) terms were used and they had been specified as the primary focus of the articles: Gene, placenta, preeclampsia, and pregnancy in the title or abstract. We also found additional MeSH terms through Cochrane Library: Transcript, sequencing, and profiling. From 687 studies retrieved from the search, only original publications that had performed high throughput sequencing of human placental tissues that reported on differentially expressed genes in pregnancies with preeclampsia were included. Two reviewers independently scrutinized the titles and abstracts before examining the eligibility of studies that met the inclusion criteria. For each study, study design, sample size, sampling type, and method for gene analysis and gene were identified. The genes listed were further analyzed with the DAVID, STRING and Cytoscape MCODE. Three original research articles involving preeclampsia comprising the datasets in gene expression were included. By combining three studies together, 250 differentially expressed genes were produced at a significance setting of *p* < 0.05. We identified candidate genes: LEP, NRIP1, SASH1, and ZADHHC8P1. Through GO analysis, we found extracellular matrix organization as the highly significant enriched ontology in a group of upregulated genes and immune process in downregulated genes. Studies on a genetic level have the potential to provide new insights into the regulation and to widen the basis for identification of changes in the mechanism of preeclampsia. Integrated bioinformatics could identify differentially expressed genes which could be candidate genes and potential pathways in preeclampsia that may improve our understanding of the cause and underlying molecular mechanisms that could be used as potential biomarkers for risk stratification and treatment.

## 1. Introduction

Preeclampsia (PE) is one of the hypertensive disorders in pregnancy characterized as hypertension developing after 20 weeks of gestation with coexistence of ≥1 of a new onset of either (1) proteinuria, (2) maternal organ dysfunction, or (3) uteroplacental dysfunction [1]. It may progress and worsen to the point of eclampsia, where the patient develops clotting abnormalities, pulmonary edema, impairment of kidney and liver function, convulsions, coma, and death. This disorder is one of the leading causes of maternal and fetal mortality and morbidity worldwide. PE is the third most prevalent cause of maternal mortality after bleeding and embolism and is responsible for around 60,000 deaths worldwide [2]. According to the World Health Organization (WHO), PE contributes to nearly 10% of all maternal mortality in Asia (WHO, 2011). PE compromises fetal growth and development, leading to small-for-gestational-age babies, premature delivery, and infant death, the pathogenesis of which is not fully understood [3].

The majority of deaths due to PE and eclampsia are preventable with timely and effective medical care. Management of women with PE aims at minimizing further pregnancy-related complications, avoiding unnecessary prematurity, and maximizing maternal and infant survival [3]. The crucial element for treatment of PE lies in the clinical categorization of patients, whether it is mild, severe PE, and worst case scenario eclampsia. For every category, different management strategies are applied. However, the clinical syndrome usually becomes apparent only after 20 weeks of gestation, which prevents early identification and may delay management. The pathogenesis of this condition is not fully understood, however it is widely accepted that vascular endothelial cell dysfunction is the final common pathway responsible for the maternal syndrome [4,5]. Even though there are many suggested mechanisms, the exact mechanism remains unclear.

Transcriptomic profiling is a gene detection technique that has been used widely for the past decade to detect all the genes within the same time-point expression information, which is suitable for differentially expressed genetic screening. Advantages of RNA sequencing over array analysis include a wider dynamic range of RNA sequencing, which is more sensitive in detecting genes with low expression level, is not probe based and, therefore, has a better genomic coverage, and lastly has the ability to decipher unannotated transcriptional activity by identifying numerous novel transcripts including protein coding and noncoding RNAs. Integrating and reanalyzing these data could provide valuable clues for new hypothesis generation and further research ideas. Many high-throughput RNA sequencing data profiling studies have been conducted on PE in recent years, and hundreds of differentially expressed genes have been identified. However, the results generated are from a single patient cohort with limited sample number. Thus, no reliable biomarkers have been identified in PE. An integrated bioinformatics method which combines several cohorts with similar expression profiling techniques could overcome this disadvantage.

In the current work, we conducted a systematic review of the available literature from several databases (PubMed, Scopus, Ovid-Medline, and GEO databases) from which a total of 61 cases of PE of placenta RNA sequencing data were extracted. Using integrated bioinformatical analysis, we developed a protein–protein interaction network and modular analysis to identify hub genes in PE. Identifying differentially expressed genes and enriching their biological functions and key pathways will provide the first step for identifying the molecular mechanisms to understanding PE.

## 2. Materials and Methods

### 2.1. Search Strategy

For a comprehensive search of genetic information, we used PubMed, Scopus, Ovid-Medline, and Gene Expression Omnibus (GEO) to identify relevant research publications with an unlimited starting publication date until 25 May 2019. The following Medical Subject Heading (MeSH) terms were used and these keywords were specified to become the primary focus of the articles: Gene, placenta, preeclampsia, pregnancy, in the title or abstract. The search strategy involved a combination (“AND”) of the following three sets of key words: (1) Gen*, (2) placent* OR pregnan*, and (3) preeclamp* OR eclamp*. Synonyms for key words were generated through MeSH terms from the Cochrane Library. Additional MeSH and text terms were identified by reviewing available review articles. These included transcript, sequencing, and profiling. Other than that, additional references were also identified after reviewing the bibliographies of the retrieved studies.

### 2.2. Inclusion Criteria

All pregnant women involved in prospective observational and case-control studies with abstracts that investigated the differentially expressed genes in placenta of preeclamptic patients were included. In addition, only studies that generate differentially expressed genes through high-throughput sequencing in the third trimester placental tissue were included to ensure homogeneity of gene expression. Due to limited resources, only manuscripts written in English were included in this review.

### 2.3. Exclusion Criteria

Publication types without primary data, such as letter to editors, editorials, case reports, conference proceedings, and narrative review articles, were excluded. This review focused on the outcome of differentially expressed genes between normotensive and third trimester preeclamptic placenta. Therefore, any studies on the first and second trimesters of pregnant women were excluded. This also meant that intervention studies where new treatments for PE were administered were excluded from consideration.

The selection criteria were used to achieve the objective of this systematic review in determining the gene expression studies of placental tissue in PE in order to determine a common expression signature and identify gene(s) and related pathways that could potentially be involved in the development of PE.

### 2.4. Screening of Articles for Eligibility

**Figure 1:**
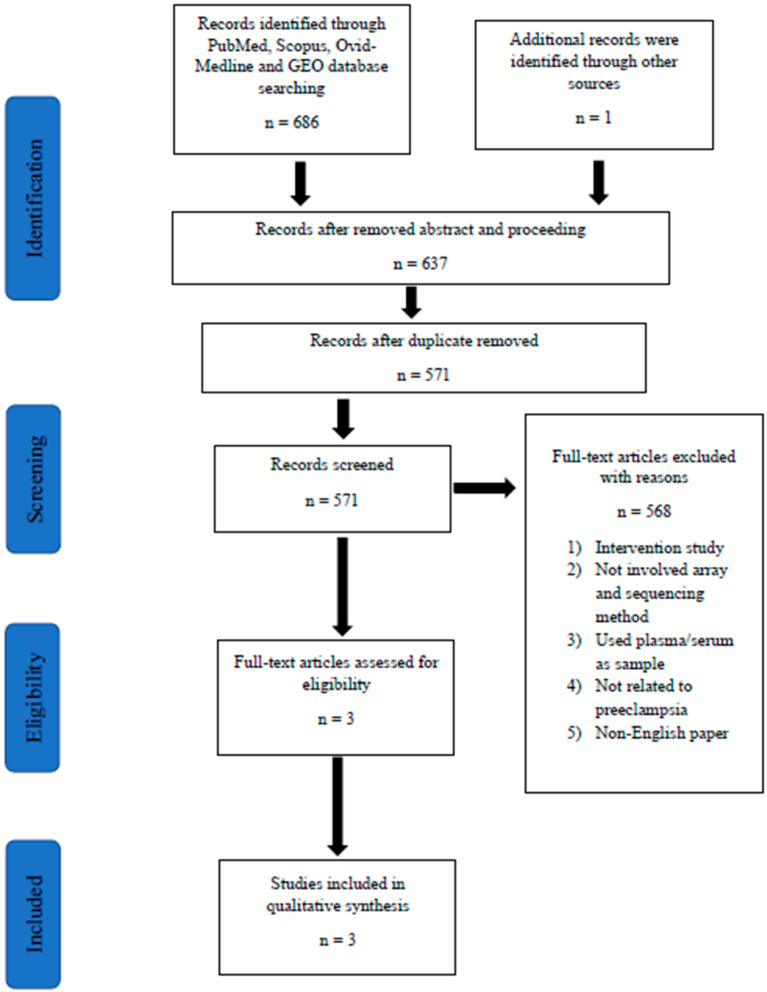
The algorithm for selection of studies in this systematic review. Abbreviation: GEO, Gene Expression Omnibus.

Retrieved articles were screened in three phases. In the first phase, any article with titles that did not match the inclusion criteria was excluded. In the second phase, the abstracts of the remaining articles were screened, and any articles that did not meet our inclusion criteria were excluded. In the final phase, full texts of the remaining articles were read and assessed thoroughly to exclude articles that did not meet our inclusion criteria or articles that fulfilled the exclusion criteria. Systematic reviews or meta-analysis were also excluded. Duplicates were removed. All the authors were involved in the selection and the data extraction phase. Any differences in opinions were resolved by discussion between the authors. In order to standardize the data collection, all data extraction was performed independently using a data collection form. Records on reasons for rejection were kept. A flow chart that summarizes the article selection process and the reasons for article exclusion is shown in Figure 1.

### 2.5. Data Extraction

The following data were extracted from the selected studies: (1) Author/study name, (2) characteristics of study design, (3) study objective, (4) study population including criteria used in defining PE and hypertension in pregnancy, (5) methodology of gene expression analysis including the characteristics of the gene expression array experiment (type of tissue, platform, details on sampling and preparation including extraction of RNA), (6) results or outcome parameters measured (genes being upregulated and downregulated), and (7) conclusion. Relevant and statistical values of gene expression were recorded. The details were extracted and are listed in Table 1.

### 2.6. Study Quality, Gene Ontology, and Pathway Analysis

The quality of each study was assessed by discussing reported details of analysis among the authors. The authors focused on the results and the reported list of gene expressions. In order to ascertain the validity of eligible studies, pairs of reviewers worked independently and with adequate reliability. For each study, bias was excluded by adhering to the inclusion criteria. The genes listed were further analyzed with the Database for Annotation, Visualization, and Integrated Discovery (DAVID). This analysis was performed to determine the cluster of genes that displayed significant functional annotation enrichment and those enriched annotations could be related to the PE, while the contribution of genes in the pathway was based on the Kyoto Encyclopedia of Genes and Genomes (KEGG) pathway, the Biological Biochemical Image database (BBID), BIOCARTA pathway database, and Reactome.

### 2.7. Protein–Protein Interaction (PPI) Network

Differentially expressed genes were further analyzed at the protein level using protein–protein interaction network functional enrichment analysis through STRING (Protein–Protein Interaction Network Functional Enrichment Analysis) (https://string-db.org/). Results from STRING were further analyzed using Cytoscape to visualize molecular interaction networks and integrating gene expression profiles. This can be useful in identifying clusters of protein interaction that are highly related to PE. The list of genes generated in the cluster was then analyzed again in DAVID for significantly enriched ontology terms.

## 3. Results

The database search identified 687 titles that were potentially relevant. The EndNote software (X9.0) by Clarivate Analytics (Philadelphia, USA) was used to identify and remove duplicates. A total of 571 articles had been retrieved for abstract review (Figure 1). Screening of titles and abstracts resulted in the selection of four potentially relevant articles for full text review. Then, 568 articles were removed based on our inclusion and exclusion criteria. Eventually, a total number of three preliminary studies were found eligible to be included in the present systematic review. All of these studies were original research articles. Homogeneity of the selected studies was ensured by adhering to the defined study design in order to prevent sampling bias, as seen in an intervention study that could interfere with the selection of cases with PE. Notably, all of the reported studies were performed in European populations: Finland, Estonia, and Norway [6,7,8]. Moreover, all these high-throughput sequencing studies included a confirmatory method to validate the RNA sequencing results using real-time polymerase chain reaction (qPCR). Sample sizes for each study varied from 8–40 samples in RNA sequencing methods [6,7,8]. Whereas for validation they ranged from 39–475 samples [6,7,8]. Table 1 highlights the characteristics of these studies.

### 3.1. Patient Recruitment and Categorization

The definitions of PE used were acceptable where appropriate categorization was achieved given that there were sufficient details on patient age, type of delivery, parity, and gestational age at delivery. The work by Kaartokallio and colleagues looked into differentially expressed genes at different gestational ages [6]. Three pools of samples were constructed based on gestational ages: (1) 38–39 weeks, (2) 34–36 weeks, and (3) at 33 weeks. In another study by Sober, the main objective was to investigate the profile of differential gene expression in prevalent adverse pregnancy outcomes at term, focusing on maternal late-onset PE, gestational diabetes, and pregnancies ending with the birth of either small-for-gestational-age (SGA) or large-for-gestational age (LGA) [7]. Lekva and colleagues categorized their patients into late-onset PE and gestational diabetes mellitus (GDM) in third trimester pregnancies [8].

### 3.2. Placenta Tissue Sampling

The definitions of PE used were acceptable and all of the studies adequately reported the type and location of tissue sampling, RNA isolation procedures, and storage protocols as well as sequencing platform used. Placenta samples obtained after caesarean delivery were included in the gene expression analysis to prevent variation on gene expression caused by the stress of labor. The section of the placenta used was part of placental parenchyma (middle region), which contain mostly villi. Two of those studies used RNAlater whereas another used nitrogen for sample preservation. Trizol reagent was used for total RNA extraction and further purified using various kits. Illumina HiSeq 2000 was used as the RNA sequencing platform for all three studies.

### 3.3. Identification of Differentially Expressed Genes in Preeclampsia

**Figure 2:**
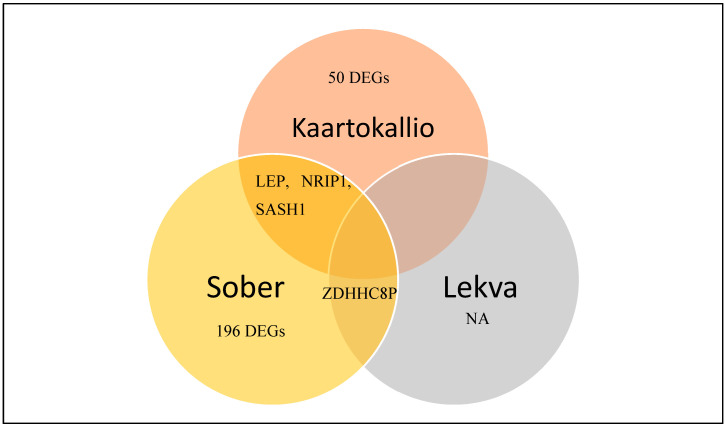
Identification of 250 commonly changed differentially expressed genes from three cohort profile datasets [6,7,8]. Different color areas represent different datasets. The overlapping areas mean the common differentially expressed genes. Statistically significant genes were defined with *p* < 0.05. (NA: Neuraminidase – unidentified gene).

NCBI-GEO is a free database of microarray/gene profile and next-generation sequencing, from which placenta tissue of PE and normal tissue gene expression profiles can be obtained. Due to the lack of standardization of sample collection and processing steps, only a handful of studies met the criteria that we set in order to be considered for analysis. The RNA sequencing data from Kaartokallio had two groups consisting of nine preeclamptic and nine normal placentae [6]. The dataset from Sober had five groups consisting of eight samples of each group: Normal placenta, late-onset PE placentae, gestational diabetes placentae, small-for-gestational-age placentae, and large-for-gestational-age placentae [7]. Lekva used 10 samples for each group: Normal placentae, gestational diabetes placentae, and preeclamptic placentae [8]. Using a *p* < 0.05 as a cut-off criterion, we extracted 53, 198, and 2 differentially expressed genes from the expression profile datasets Kaartokallio, Sober, and Lekva, respectively. After integrated bioinformatics analysis, a total of 250 consistently expressed genes were identified from the three profile datasets (Figure 2), including 228 upregulated genes and 22 downregulated genes in preeclamptic placenta tissue, compared to normal placenta tissues (Table 2).

### 3.4. Differentially Expressed Genes’ Ontology Analysis in Preeclampsia

**Figure 3:**
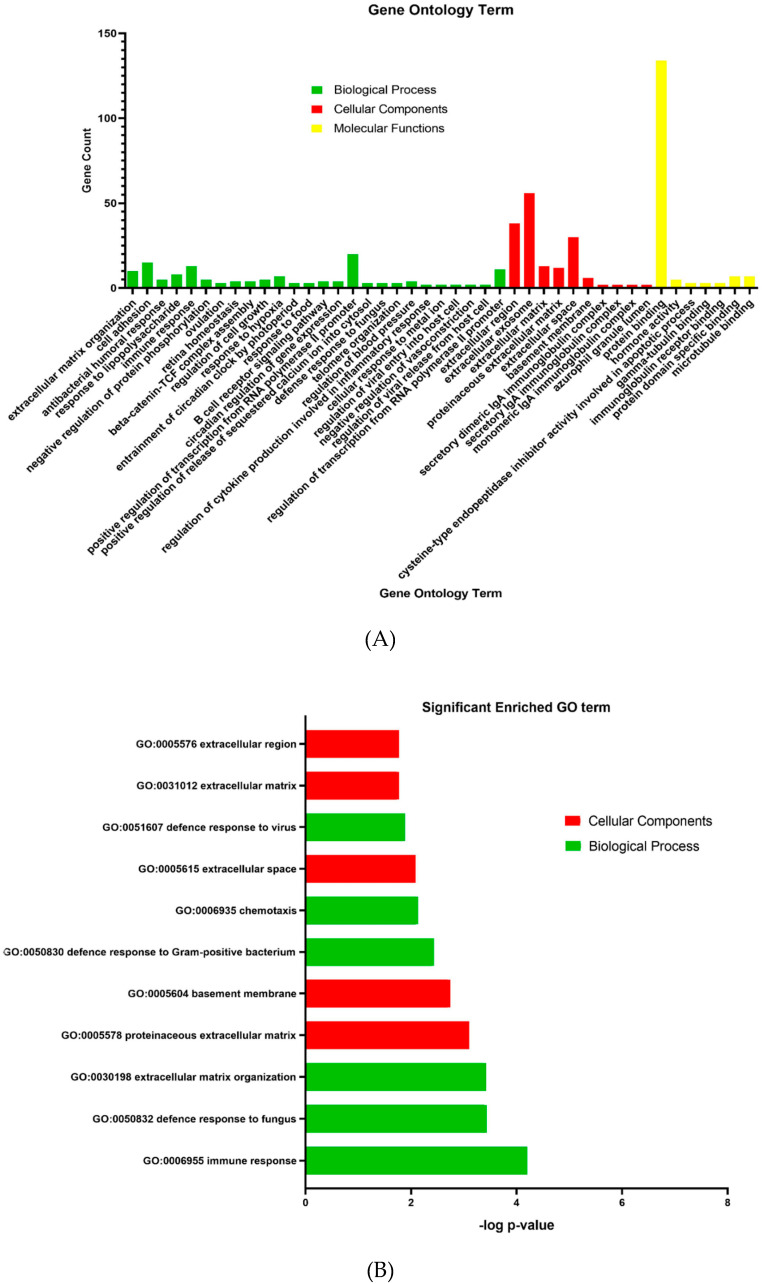
Gene ontology analysis and significant enriched GO terms of differentially expressed genes in placenta tissue of patients with preeclampsia. (**A**) GO analysis classification of upregulated and downregulated differentially expressed genes into three groups (i.e., biological process, cellular components, and molecular function). (**B**) Significantly enriched GO terms of upregulated and downregulated differentially expressed genes in patients with preeclampsia based on their functions (11 terms).

Candidate differentially expressed genes (DEGs) functions and pathways enrichment were analyzed using DAVID (https://david.ncifcrf.gov/home.jsp). A p-value of <0.05 was used as a cut-off criterion. DEGs’ gene ontology analysis (GO) was performed on DAVID (Tables S1 and S2). The DEGs were classified into three functional groups: (1) Molecular function, (2) biological process, and (3) cellular component (Figure 3). As shown in Table 3, in the biological process group, upregulated genes mainly enriched in extracellular matrix organization. In the cellular components group, upregulated genes mainly enriched in the proteinaceous extracellular matrix, extracellular region, and basement membrane, whereas downregulated genes in this category were mainly enriched in extracellular space and extracellular matrix.

### 3.5. Signalling Pathway Enrichment Analysis

**Figure 4:**
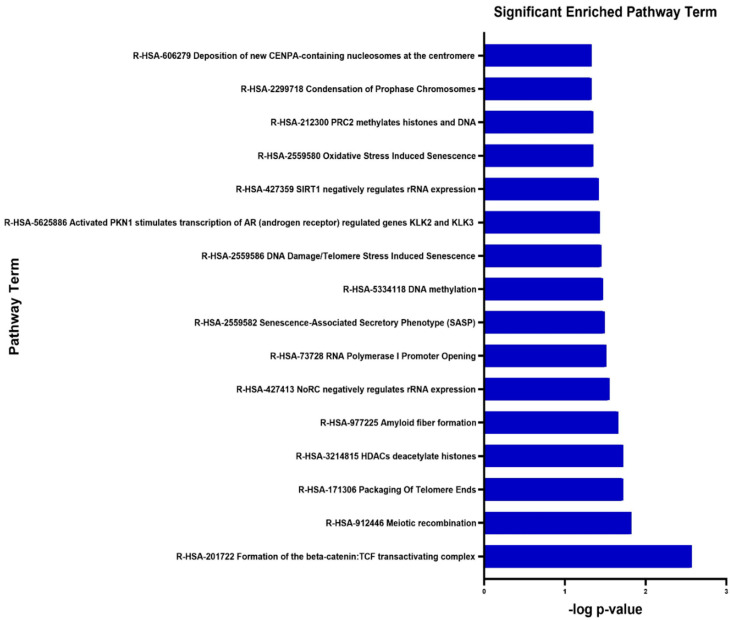
Significantly enriched pathway terms of upregulated differentially expressed genes in placenta tissue of patients with preeclampsia. Differentially expressed genes’ functional and signaling pathway enrichment were conducted using online websites for KEGG PATHWAY, BBID, BIOCARTA and Reactome.

The DEGs’ functional and signaling pathway enrichment were conducted using the online website of DAVID with integrated KEGG PATHWAY, BBID, BIOCARTA, and Reactome. The upregulated genes mainly enriched in formation of beta-catenin: TCF transactivating complex (R-HSA-201722), meiotic recombination (R-HSA-912446), and packaging of telomere ends (R-HSA-171206) (Figure 4, Table 4). There were no significantly enriched pathways related to downregulated genes.

### 3.6. Key Candidate Genes’ and Pathways’ Identification with Differentially Expressed Genes’ Protein–Protein Interaction Network (PPI) and Modular Analysis

**Figure 5:**
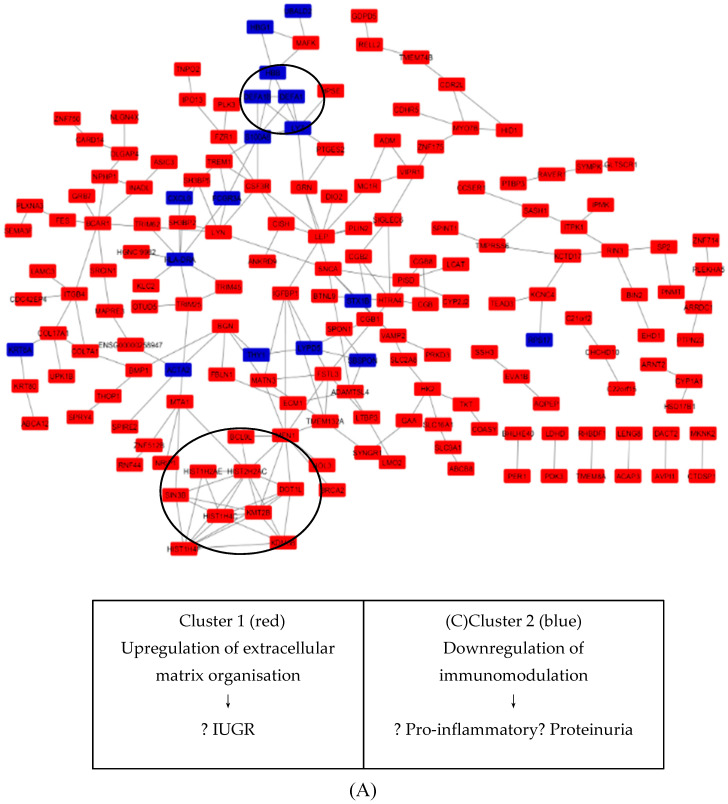

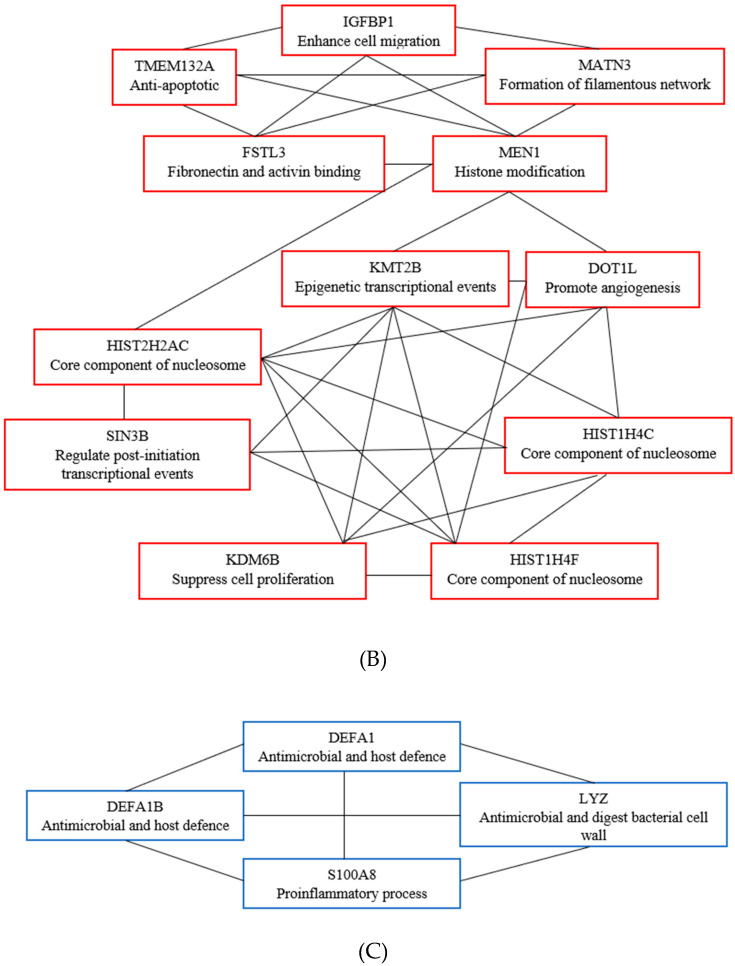
STRING analysis DEGs protein–protein interaction (PPI) network and modular analysis. (**A**) Using STRING online database, total of 250 DEGs (228 upregulated in red standing and 22 downregulated in blue standing) were filtered into the DEGs’ PPI network complex. The two highlighted circle areas are the two most significant clusters identified through MCODE. (**B**) Cluster 1 consists of 12 nodules and 32 edges. (**C**) Cluster 2 consists of 4 nodules and 6 edges.

Using the STRING online database (available online at http://string-db.org), a total of 250 DEGs (22 upregulated and 228 downregulated genes) of the 250 commonly altered DEGs were filtered into the DEGs’ PPI network complex, containing 237 nodes and 217 edges (Figure 5A), while 13 DEGs did not fall into the DEGs’ PPI network. According to the degree of importance, we transferred results from STRING to Cytoscape. Through Cytoscape MCODE, we chose 2 significant modules from the PPI network complex for further analysis. Function annotation clustering showed that Cluster 1 consisted of 12 nodes and 32 edges (Figure 5B), which were mainly associated with PKMTs methylate histone lysines pathway, formation of beta catenin: Transactivating complex pathway and telomere organization (Table 5), while Cluster 2 consisted of 4 nodes and 6 edges (Figure 5C), which are mainly associated with defense response to fungus, defense response to Gram-positive bacterium, and extracellular space (Table 6).

## 4. Discussion

In the past several decades, numerous basic and clinical studies have been conducted in an attempt to reveal the causes and underlying mechanisms of PE development and progression to eclampsia [4,9,10,11,12]. However, most studies were focused on single molecular event or the result of single cohort studies. At present, the mechanism of PE still remains unclear. The placenta is the key organ involved in PE. We attempted to improve our understanding of the molecular mechanisms at play by identifying DEGs from three study datasets. Step one of the analysis involved pooling the datasets of the relevant studies. In step two, DEGs were classified into three major functional groups based on GO terms (Figures S1 and S2) using multiple approaches. In step three, the up- and downregulated DEGs were further clustered based on functional and signaling pathways with significant enrichment analysis. Step four was the development of PPI network complex of the DEGs, where, finally, the two most significant modules were filtered.

In this review, the biological process GO term, extracellular matrix organization, revealed the highest relation to all upregulated DEGs identified in Cluster 1 of the PPI network. This biological process influences many cell behaviors such as proliferation, adhesion, migration, and regulation of cell differentiation and death. Dysregulation of the extracellular matrix can lead to disruption of cell proliferation and invasion, failure of cell death, and loss of cell differentiation [13,14].

Through integrated bioinformatical analysis, we identified 16 central node/genes. Among them, the first module or cluster (Figure 5B) consisted of 12 upregulated genes: TMEM132A, FSTL3, MEN1, MATN3, IGFBP1, KMT2B, HIST2H2AC, SIN3B, KDM6B, HIST1H4F, HIST1H4C, and DOT1L. Genes in this cluster appeared to be crucial for extracellular matrix organization. Two of the genes in this cluster, FSTL3 and IGFB1, have been reported to play a role in PE. The expression of FSTL3 was increased across gestational trimester in PE. One of the suggested mechanisms involved in PE is hypoxia. Studies have reported that hypoxia enhances the expression of FSTL3 in term human trophoblast [15]. Another gene, IGFBP1, is generally involved in cell migration and metabolism, where insufficient branched-chain amino acid supply to uterine decidual cells decreases IGFBP1 expression and affects the migration of extravillous trophoblast (EVT) in early pregnancy [16]. Another study reported that hyperphosphorylation of IGFBP1 in decidualized stromal mesenchymal decidua basalis is increased in intrauterine growth restrictions (IUGR) pregnancies [17].

The second cluster of the PPI network consisted of 4 genes which were DEFA1, DEFA1B, LYZ, and S100A8 (Figure 5C). Genes in this cluster suggested a mechanism involving maintaining a balance in immunomodulation between the maternal and fetal interface. In a normal pregnancy, the maternal immune system attempts to balance the pro-inflammatory and anti-inflammatory proteins in order to adapt to the semi-allogeneic fetus for successful pregnancies. The disruption of this process may cause the imbalance of immune cell and cytokines production. A study by Ma and colleagues showed that there were significantly increased pro-inflammatory cytokines IL-1β, IL-6, IL-7, IL-8, IL-17a, MIP-1β, and MCP1 in serum of PE patients when compared to normal patients [18]. LYZ and S100A8 have both been reported to be involved in PE. LYZ encodes for lysozyme that cleaves beta glycosidic linkages between N-acetylmuramic acid and N-acetylglucosamine in bacterial cell wall peptidoglycan. Beta-hexosaminidase, a type of lysozyme, was reduced in the urine of preeclamptic patients. However, this beta-hexosaminidase is associated with proteinuria, not hypertension. Because of this distinction, it could be used to further our understanding of the mechanism of proteinuria which occurs in PE. S100A8, on the other hand, generally has a prominent role in the regulation of inflammatory processes and immune response. S100A8 enhances pro-inflammatory activity which includes recruitment of leukocytes, production of cytokines and chemokines, and regulation of leukocyte adhesion and migration. S100A8, also known as calprotectin, has been reported to be significantly increased among preeclamptic patients as the pregnancy progresses.

The biological pathway generated from this analysis enriched into the formation of the β-catenin: TCF transactivating complex pathway (R-GGA-201722). β-catenin is a dual-function protein which is involved in both the regulation and coordination of cell–cell adhesion and gene transcription. Cell adhesion events are essential for normal placenta development. The β-catenin: TCF transactivating complex pathway is closely related to Wnt signaling through the interaction with TCF/LEF transcription factors. BCL9L, HIST1H2AE, HIST1H4C, HIST1H4F, HIST2H2AC, and MEN1 are genes involved in this pathway.

Even though each cohort had a predetermined and different p-value, we set to include only differentially expressed genes where the FDR was reported. Interestingly, the recent studies reported identification of DEGs in PE. We identified four candidate genes from the three patient cohorts: LEP, NRIP1, SASH1 [6,7], and ZDHHC8P1 [7,8]. Leptin (LEP) is a gene that has been frequently investigated in PE. As reported by previous systematic review studies, LEP has been identified as a meta signature of third trimester [19] and highly associated with PE [20]. Generally, leptin is a hormone produced by adipose cells that is crucial in the regulation of energy consumption, food intake, and adiposity [21]. During pregnancy, leptin is produced in syncytiotrophoblast and endothelial cells of the placenta [22,23]. Leptin is known to have a role in pregnancy by activating the leptin signaling pathway in trophoblasts to promote placental growth and has an anti-apoptotic role as well [24]. During the early stages of pregnancy, LEP regulates angiogenesis, growth, and immunomodulation [25,26,27]. The disruption of these processes may lead to faulty implantation of blastocyst, which may cause a miscarriage. A meta-analysis revealed that LEP interacted with CREBBP protein, which is a key protein–protein interaction in PE [28]. There have been studies reporting that higher serum leptin is observed in preeclamptic patients compared to normal pregnancies [29,30]. In addition to this, serum LEP has been reported to be higher in early-onset PE compared to late-onset PE [31]. This, however, contrasts with others who did not find any correlation between leptin expression with PE [32]. The function of leptin and its involvement in the mechanism of PE is still unclear. Hence, further investigation should be conducted to find the exact role of leptin on mechanism in PE.

Nuclear receptor-interacting protein 1 (NRIP1) is another gene that has been differentially expressed and upregulated in placenta of preeclamptic patients [6,7]. NRIP1 is also known as receptor-interacting protein 140 (RP140). It functions as a co-regulator either by co-activating or co-repressing for numerous nuclear receptor transcription factors. Transcription factors such as thyroid hormone receptor α (TRα), estrogen-related receptor α (ERRα), ERRγ, liver X receptor (LXR), peroxisome proliferator activator receptor α (PPARα), PPARγ, and E2F transcription factor 1 (E2F1) are co-repressed by NRIP1 [33]. NRIP1 acts as co-activator in the regulation of mammary gland development, circadian rhythms, inflammation, and ovulation, and is poorly understood [33]. It has been identified as one of two genes that were specifically expressed in PE across three transcriptomic platforms (microarray, RNA-Seq, and PAS-Seq) [34]. The other being FLT1 gene. This expression aligns with NRIP1 function on ovarian fertility and maintenance of pregnancy state [35]. NRIP1 is also reported to be prominently related to the pathogenesis of obesity [36]. There is a possibility that upregulated NRIP1 co-activates anti-angiogenic factors to promote the endothelial dysfunction in PE.

SASH1 is known as a novel candidate of tumor suppressor gene. Some studies have reported that reduced SASH1 expression will inversely increase the tumor proliferation, invasion, and metastasis [37,38]. SASH1 encodes a scaffold protein involved in the toll-like receptor 4, TLR4 signaling pathway that regulates the cascade of cytokine production and endothelial cell migration in response to invading pathogens [39]. SASH1 is shown to be downregulated in breast cancer [40] and, when highly expressed, SASH1 can inhibit proliferation, invasion, and endothelial mesenchymal transition in other malignancies such as hepatocellular carcinoma and gastric cancer [41,42]. ZDHHC8P1 is upregulated and differentially expressed in two preeclamptic patient cohorts. It is a pseudogene in which a phenotype has yet to be reported and the gene’s in vivo function is still unknown. ZDHHC8P1 is highly expressed in the prostate, ovary, and placenta. One recent study reported that ZDHHC8P1 promotes the progression and metastasis of colorectal cancer by targeting miR-34a [43]. Its role in the mechanism of PE has yet to be studied.

The fact that there is no database for the development of PE, as seen with solid tumors, for example, is a major limiting factor in this review. The major obstacles in the initial filtering and quality assessment of studies were the incomplete listing of differentially expressed genes, sorting through different study structures, and understanding the diversity in statistical procedures used. Using a strict inclusion criterion, only high-throughput gene sequencing results were considered for analysis. Many studies have reported that early- and late-onset PE have different pathophysiologies. As early or first trimestral data were excluded from the current analysis, we could not draw any firm conclusions with regards to genetic predisposition for PE and it remains an interesting comparative analysis to perform in the future. Because the data we retrieved from this studies did not include raw data of normal placenta, the activation or deactivation of certain genes could not be ascertained. However, this is an area of great interest for exploration by using sophisticated RNA interference strategies.

## 5. Conclusions

PE constitutes a condition that develops and worsens over the course of the growing fetus. However, not all women are affected. This signifies that there are differing sets of epigenetic and genetic events leading to alteration of gene expression at transcriptional and translational levels, involving multiple functional signaling pathways. Imbalance of angiogenesis and hypoxia have been identified as key factors involved in PE [44,45]. In the current work, extracellular matrix organization and immune process were found to be highly significant biological processes in PE. This could relate to the fact that all cohorts in this review were of late-onset PE. The characteristics of PE cannot be explained only by gene expression profiles. Narrowing the field of possible genes would allow further research for future validation at the proteomic level.

## Figures and Tables

**Table 1:** Summary of the selected studies.

Title and Reference	Year	Study Design	Study Population	Findings	
				Gene Expression Analysis Parameters	Genes Upregulated and Down Regulated
Gene expression profiling of pre-eclamptic placentae by RNA sequencing (Kaartokallio, Cervera et al. 2015)	2015	Case-control study	RNASeq (n = 18, 3 groups) qPCR (n = 39, 3 groups)	RNA Sequencing (Illumina) was performed on placenta at different gestational age Gene extraction by miRVana (RNAseq), PureLink (qPCR) Sampling of 9-site on placenta Differentiation threshold: FDR (<0.05) via CummerRband	Upregulated 32 genes Downregulated 21 genes
Extensive shift in placental transcriptome profile in preeclampsia and placental origin of adverse pregnancy outcomes (Sõber, Reiman et al. 2015)	2015	Case-control study	RNASeq (n = 40, 5 groups), qPCR (n = 120, 5 groups)	RNA sequencing (Illumina) on LO-PE, Gestational Diabetes Gene extraction by RNAeasyMinElute (RNAseq), Nucleospin II (qPCR) Sampling of middle region of placenta Differentiation threshold: DESeq FDR < 0.1, DESeq2 FDR < 0.05	Upregulated 215 genes Downregulated 10 genes
Gene expression in term placentas is regulated more by spinal or epidural anaesthesia than by late-onset preeclampsia or gestational diabetes mellitus (Lekva, Lyle et al. 2016)	2016	Case-control study	RNASeq (n = 30, 3 groups), qPCR (n = 475)	RNA sequencing on (Illumina LO-PE, GDM, cofounding variables such as type of delivery Gene extraction by RNAEasy Microkit (RNASeq, qPCR) Sampling of placental parenchyma Differentiation threshold: DESeq2 FDR < 0.1	Upregulated 1 gene Downregulated 4 genes

**Table 2:** The 250 differentially expressed genes (DEGs) were identified from three profile datasets, including 228 upregulated genes and 22 downregulated genes in the placenta tissue of preeclampsia compared to normal placenta tissues.

Differentially Expressed Gene	Gene Name
Upregulated	ABCA12, ABCB8, ACAP3, ADAMTSL4, ADM, AHDC1, ANKRD33, ANKRD9, APBA3, LVRN, ARMS2, ARNT2, ARRDC1, ASIC3, AVPI1, BCAR1, BCL9L, BGN, BHLHE40, BIN2, BMP1, BRCA2, BTNL9, ADIRF, C10orf54, C12orf75, C15orf39, NATD1, C1QTNF1, C21orf2, C22orf15, C7orf43, CARD14, CCSAP, CCSER1, CD320, CDC42EP4, CDHR5, CDR2L, CGB1, CGB2, CGB8, CHCHD10, CISH, CLPTM1, CNTNAP3B, COASY, COL17A1, COL7A1, CORO2A, CPZ, CRLF1, CRTC2, CSF3R, DDX43P1, CTDSP1, CYP2J2, CYP51A1P3, DACT2, DDX60L, DIO2, DLGAP4, DLX3, DLX4, DNAH11, DOT1L, DPPA4, ECM1, EHD1, EMC10, EVA1B, FAM109A, FAM160B2, FBLN1, FER1L4, FES, FOXO6, FSTL3, FZR1, GAA, GDPD5, GLTSCR1, GRAMD1A, GRB7, GRINA, GRN, GUSBP3, HID1, HIST1H2AE, HIST1H4C, HIST1H4F, HIST2H2AC, HK2, HPSE, HSD11B2, HSD17B1, HSD17B1P1, HTRA4, IGFBP1, IGHA1, IGHA2, IPMK, IPO13, ITGB4, ITPK1, ITPK1-AS1, KCNC4, KCTD17, KDM6B, KIAA1211, KIAA1522, KLC2, KRT80, LAMC3, LCAT, LDHD, LENG8, LEP, LMO2, LRCH4, LTBP3, LTBR, LYN, MAFK, MAGEA11, MAP1S, MAPRE3, MATN3 MC1R, MEN1, MIR205HG, MKNK2, MTA1, MYO7B, NDRG1, NLGN4X, NOL3, NPHP1, NRIP1, OTUD5, P2RY6, PDK3, PER1, PIGT, PIM3, GSAP, PISD, PLEKHA5, PLEKHA7, PLIN2, PLK3, PLXNA3, PNMT, PRKD3, PTBP3, PTGES2, PTPN23, RAB4B-EGLN2, RAVER1, RELL2, RFX1, RHBDF1, RIN3, RNF44, RTEL1-TNFRSF6B, SASH1, SCAF1, SEMA3F, SERPINB9, SH3BP2, SH3BP5, SH3GLB2, SIGLEC6, SIN3B, SLC16A1, SLC25A35, SLC26A11, SLC2A8, SLC9A1, SLCO3A1 SMOC1, SNCA, SNX8, SP2, SPINT1, SPIRE2, SPON1, SPRY4, SRCIN1, SSH3, ST8SIA4, STX1B, SYMPK, SYNGR1, TCTEX1D4, TEAD3, TET3, THOP1, TKT, TMEM132A, TMEM74B, TMEM8A, TMPRSS6, TNPO2 TPBG, TREM1, TRIM25, TRIM45, TRIM62, TUBB3, UBALD1, UBALD2, ULK1, UPK1B, VAMP2, VIPR1, KMT2B, ZDHHC8P1, ZNF124, ZNF175, ZNF512B, ZNF714, ZNF750, ZNF76, ZNF90, CGB3, PATJ, NECTIN4
Downregulated	ACTA2, CXCL9, CYP1A1, DEFA1, DEFA1B, FCGR3A, HBB, HBG1, HLA-DRA, KRT6A, LGALS14, LOC100129345, LYPD5, LYZ, MAGEA4, NCMAP, PSG10P, RPS17, S100A8, SBSPON, THY1, ZDHHC1

**Table 3:** The significantly enriched analysis of differentially expressed genes in preeclampsia.

Term	Description	Count	p-Value	-Log_10_ p Value
Up-regulated				
GO:0030198 BP	extracellular matrix organization	10	3.80 × 10^−4^	3.42
GO:0005578 CC	proteinaceous extracellular matrix	11	7.80 × 10^−4^	3.10
GO:0005604 CC	basement membrane	6	1.80 × 10^−4^	2.74
GO:0031012 CC	extracellular matrix	9	1.70 × 10^−4^	1.77
Downregulated				
GO:0006955 BP	immune response	6	6.30 × 10^−5^	4.20
GO:0050832 BP	defense response to fungus	3	3.70 × 10^−4^	3.43
GO:0050830 BP	defense response to Gram-positive bacterium	3	3.70 × 10^−3^	2.43
GO:0006935 BP	chemotaxis	3	7.40 × 10^−3^	2.13
GO:0005615 CC	extracellular space	6	8.30 × 10^−3^	2.08
GO:0051607 BP	defense response to virus	3	1.30 × 10^−2^	1.88
GO:0005576 CC	extracellular region	6	1.70 × 10^−2^	1.77

**Table 4:** Signaling pathway enrichment analysis of upregulated differentially expressed genes’ function in placenta tissue of patients with preeclampsia.

Pathway	Name	Genes	p-Value	-Log_10_ p Value
R-HSA-201722	Formation of the beta-catenin: TCF transactivating complex	BCL9L, HIST1H2AE, HIST1H4C, HIST1H4F, HIST2H2AC, MEN1	2.70 × 10^−3^	2.57
R-HSA-912446	Meiotic recombination	BRCA2, HIST1H2AE, HIST1H4C, HIST1H4F, HIST2H2AC	1.50 × 10^−2^	1.82
R-HSA-171306	Packaging Of Telomere Ends	HIST1H2AE, HIST1H4C, HIST1H4F, HIST2H2AC	1.90 × 10^−2^	1.72
R-HSA-3214815	HDACs deacetylate histones	HIST1H2AE HIST1H4C HIST1H4F HIST2H2AC MTA1	1.90 × 10^−2^	1.72
R-HSA-977225	Amyloid fibre formation	HIST1H2AE HIST1H4C HIST1H4F HIST2H2AC SNCA	2.20 × 10^−2^	1.66
R-HSA-427413	NoRC negatively regulates rRNA expression	SIN3B HIST1H2AE HIST1H4C HIST1H4F HIST2H2AC	2.80 × 10^−2^	1.55
R-HSA-73728	RNA Polymerase I Promoter Opening	HIST1H2AE HIST1H4C HIST1H4F HIST2H2AC	3.10 × 10^−2^	1.51
R-HSA-2559582	Senescence-Associated Secretory Phenotype (SASP)	FZR1 HIST1H2AE HIST1H4C HIST1H4F HIST2H2AC	3.20 × 10^−2^	1.49
R-HSA-5334118	DNA methylation	HIST1H2AE HIST1H4C HIST1H4F HIST2H2AC	3.40 × 10^−2^	1.47
R-HSA-2559586	DNA Damage/Telomere Stress Induced Senescence	HIST1H2AE HIST1H4C HIST1H4F HIST2H2AC	3.50 × 10^−2^	1.45
R-HSA-5625886	Activated PKN1 stimulates transcription of AR (androgen receptor) regulated genes KLK2 and KLK3	HIST1H2AE HIST1H4C HIST1H4F HIST2H2AC	3.70 × 10^−2^	1.43
R-HSA-427359	SIRT1 negatively regulates rRNA expression	HIST1H2AE HIST1H4C HIST1H4F HIST2H2AC	3.80 × 10^−2^	1.42
R-HSA-2559580	Oxidative Stress Induced Senescence	KDM6B HIST1H2AE HIST1H4C HIST1H4F HIST2H2AC	4.50 × 10^−2^	1.35
R-HSA-212300	PRC2 methylates histones and DNA	HIST1H2AE HIST1H4C HIST1H4F HIST2H2AC	4.50 × 10^−2^	1.35
R-HSA-2299718	Condensation of Prophase Chromosomes	HIST1H2AE HIST1H4C HIST1H4F HIST2H2AC	4.70 × 10^−2^	1.33
R-HSA-606279	Deposition of new CENPA-containing nucleosomes at the centromere	HIST1H2AE HIST1H4C HIST1H4F HIST2H2AC	4.70 × 10^−2^	1.33

**Table 5:** Functional annotation clustering on cluster 1.

Term	Description	Number of Genes	p-Value
R-HSA-3214841	PKMTs methylate histone lysines	4	3.88 × 10^−5^
R-HSA-201722	Formation of the beta-catenin:TCF transactivating complex	4	1.01 × 10^−4^
GO:0032200	Telomere organization	3	1.11 × 10^−4^
R-HSA-427413	NoRC negatively regulates rRNA expression	4	7.51 × 10^−5^
R-HSA-2559580	Oxidative Stress Induced Senescence	4	2.72 × 10^−4^
GO:1904837	Beta-catenin-TCF complex assembly	3	2.85 × 10^−4^
GO:0000786	Nucleosome	3	1.4 × 10^−3^
R-HSA-171306	Packaging of Telomere Ends	3	1.4 × 10^−3^
GO:0043234	Protein complex	4	1.65 × 10^−3^
R-HSA-73728	RNA Polymerase I Promoter Opening	3	2.06 × 10^−3^
R-HSA-5334118	DNA methylation	3	2.19 × 10^−3^
R-HSA-2559586	DNA Damage/Telomere Stress Induced Senescence	3	2.26 × 10^−3^
R-HSA-5625886	Activated PKN1 stimulates transcription of AR (androgen receptor) regulated genes KLK2 and KLK3	3	2.32 × 10^−3^
R-HSA-427359	SIRT1 negatively regulates rRNA expression	3	2.39 × 10^−3^
GO:0000784	Nuclear chromosome, telomeric region	3	2.66 × 10^−3^
R-HSA-212300	PRC2 methylates histones and DNA	3	2.75 × 10^−3^
R-HSA-2299718	Condensation of Prophase Chromosomes	3	2.83 × 10^−3^
R-HSA-606279	Deposition of new CENPA-containing nucleosomes at the centromere	3	2.83 × 10^−3^
R-HSA-3214858	RMTs methylate histone arginines	3	3.06 × 10^−3^
R-HSA-1221632	Meiotic synapsis	3	3.22 × 10^−3^
hsa05322	Systemic lupus erythematosus	3	3.62 × 10^−3^
R-HSA-912446	Meiotic recombination	3	3.89 × 10^−3^
R-HSA-73777	-	3	4.16 × 10^−3^
R-HSA-5250924	B-WICH complex positively regulates rRNA expression	3	4.25 × 10^−3^
R-HSA-3214815	HDACs deacetylate histones	3	4.53 × 10^−3^
R-HSA-977225	Amyloid fiber formation	3	4.91 × 10^−3^
R-HSA-5578749	Transcriptional regulation by small RNAs	3	5.72 × 10^−3^
R-HSA-2559582	Senescence-Associated Secretory Phenotype (SASP)	3	6.15 × 10^−3^
hsa05034	Alcoholism	3	6.26 × 10^−3^
R-HSA-5617472	Activation of anterior HOX genes in hindbrain development during early embryogenesis	3	7.52 × 10^−3^
R-HSA-3214847	HATs acetylate histones	3	1.01 × 10^−2^
GO:0003677	DNA binding	5	1.24 × 10^−2^
GO:0046982	protein heterodimerization activity	3	2.94 × 10^−2^

**Table 6:** Functional annotation clustering on cluster 2.

Term	Description	Number of Genes	p-Value
GO:0050832	Defense response to fungus	3	7.46 × 10^−6^
GO:0050830	Defense response to Gram-positive bacterium	3	7.57 × 10^−5^
GO:0005615	Extracellular space	4	4.03 × 10^−4^
GO:0005576	Extracellular region	4	6.88 × 10^−4^
GO:0045087	Innate immune response	3	1.92 × 10^−3^
GO:0070062	Extracellular exosome	4	3.67 × 10^−3^

## Supplementary Materials

The following are available online at https://www.mdpi.com/2079-7737/9/4/62/s1, Figure S1: Gene Ontology Term of upregulated differentially expressed genes. Figure S2: Gene Ontology Term of downregulated differentially expressed genes. Table S1: Gene Ontology of Upregulated Genes. Table S2: Gene Ontology of Downregulated Genes.
